# Durable patient-reported outcomes following 60-day percutaneous peripheral nerve stimulation (PNS) of the medial branch nerves

**DOI:** 10.1016/j.inpm.2023.100243

**Published:** 2023-03-13

**Authors:** Christopher A. Gilmore, Timothy R. Deer, Mehul J. Desai, Thomas J. Hopkins, Sean Li, Michael J. DePalma, Steven P. Cohen, Meredith J. McGee, Joseph W. Boggs

**Affiliations:** a Center for Clinical Research, Carolinas Pain Institute, Winston-Salem, NC, USA; b Spine & Nerve Centers of the Virginias, Charleston, WV, USA; c International Spine, Pain & Performance Center, Washington, DC, USA; d Duke University, Durham, NC, USA; e Premier Pain Centers, Shrewsbury, NJ, USA; f Virginia iSpine Physicians, Richmond, VA, USA; g Johns Hopkins School of Medicine, Baltimore, MD, USA; h SPR Therapeutics, Inc, Cleveland, OH, USA

**Keywords:** Chronic low back pain, Percutaneous peripheral nerve stimulation, Neuromodulation, Non-opioid, Disability, Pain management

## Abstract

**Background:**

Chronic low back pain (CLBP) is often associated with clinical evidence of central nervous system sensitization and finding a clear source of nociceptive input can be challenging. Conventional therapies targeting peripheral spinal pain structures can fail to address centrally-mediated, underlying causes of pain. Sixty-day percutaneous peripheral nerve stimulation (PNS) applied to the lumbar medial branch nerves is a non-surgical, non-opioid treatment that may restore the balance of peripheral inputs to the central nervous system and reverse maladaptive changes in central pain processing. As a minimally invasive, non-destructive treatment, percutaneous PNS was designed to be used earlier in the treatment continuum than radiofrequency ablation or permanently-implanted neurostimulation systems.

**Objective:**

The objective of this clinical trial was to characterize the durability of responses to medial branch PNS in a prospective multicenter case series study of CLBP patients recalcitrant to multiple non-surgical treatments.

**Design:**

Prospective, multicenter clinical trial.

**Population:**

Adults with CLBP without radicular leg pain who had previously failed multiple types of conventional treatments.

**Intervention:**

Sixty-day percutaneous PNS applied to the lumbar medial branch nerves.

**Methods:**

Percutaneous PNS leads were implanted under image guidance (ultrasound and/or fluoroscopy) and treatment was applied for up to 60 days, after which the leads were removed. Participants were followed through 14 months (12 months after the 2-month PNS treatment). Prospectively-defined endpoints included assessments of pain intensity, disability, pain interference, health-related quality of life, depression, and patient global impression of change.

**Results:**

Treatment of CLBP with 60-day percutaneous PNS treatment produced clinically meaningful improvements in average pain intensity, disability, and/or pain interference for a majority of participants through the entire 14 month follow up period without requiring permanent system implantation. The proportion of participants experiencing clinically meaningful improvement in at least one outcome (pain intensiy, disability, pain interference) with PNS was 91% after 2 months, 79% at 5 months, 73% at 8 months, 75% at 11 months, and 77% at 14 months. There were no serious or unanticipated study-related adverse events.

**Conclusion:**

This prospective multicenter clinical trial demonstrates the clinical utility of percutaneous PNS when applied to the medial branch nerves for the treatment of chronic low back pain recalcitrant to non-surgical treatments. Given the minimally invasive nature of percutaneous PNS and the significant benefits experienced by participants, percutaneous PNS provides a safe and effective first-line neuromodulation treatment for patients with CLBP that may obviate the need for neuroablative procedures or permanent neurostimulation system implantation.

## 1 Introduction

Chronic low back pain (CLBP) is the leading cause of disability worldwide and is associated with significant societal and economic burdens [1,2]. CLBP is often associated with a significant centrally-mediated component, where the pain is maintained, at least in part, by sensitization of circuits in the central nervous system (CNS; *e.g.*, spinal cord and brain) [[3], [4], [5], [6], [7]] and there is well-documented evidence of biomarker changes in central pain processing [6,[8], [9], [10], [11]]. Central sensitization is believed to occur due to an imbalance of sensory input to the CNS following injuries or pathology in the periphery (*e.g.,* elevated nociceptive input results in inflammatory neurophysiological changes and sensitization of the periphery and CNS processing) or from psychological inciting events [[12], [13], [14]]. Evidence of these changes in central pain processing and abnormal functional connectivity between the medial prefrontal cortex and other brain regions have been confirmed in patients with CLBP compared to healthy controls [9,10].

Finding a clear source of nociceptive input or damage to the somatosensory system can be challenging for patients with CLBP, since the pain may be widespread and/or non-specific, and reinforced by symptoms of central nervous system sensitization [11,15]. Given the involvement of central sensitization and changes in CNS pain processing, it is not surprising that treatment of CLBP has historically been very difficult. While conventional pain management approaches for CLBP may work in carefully selected patients, some approaches are not effective long-term or have significant side effects (*e.g.*, opioids, or non-opioids) and other treatments are associated with poor compliance or diminishing returns (*e.g.*, physical therapy, injections, radiofrequency ablation) [[16], [17], [18], [19], [20], [21], [22]]. Therapies that seek to mask the pain rather than address the potentially centrally mediated, underlying causes of pain typically require retreatment or continuous use to provide a sufficient analgesic effect, which may accommodate or wane with time [19,23,24]. Some have suggested that rather than attempting (often futilely) to categorize CLBP pathologies to treat specific symptoms, treatments should be developed that target the underlying condition of central sensitization as variable symptom presentation may be mediated by the same central processes [25].

Percutaneous peripheral nerve stimulation (PNS) is a temporary (60-day) treatment designed to generate proprioceptive afferent signals believed to restore the balance of peripheral inputs to the central nervous system and reverse maladaptive changes in central pain processing [26]. Given the relationship between degenerative anatomical and neural signaling changes and maladaptive central plasticity in CLBP, percutaneous PNS applied to the lumbar medial branch nerves seeks to address the underlying centrally-mediated component through a minimally-invasive, temporary neuromodulatory approach. Recent studies demonstrating tolerance within 1 year for the therapeutic effects of spinal cord stimulation suggest that temporary neuromodulatory therapies may exert similar modulatory and analgesic effects, with superior risk-benefit and cost-effectiveness ratios [27]. Although percutaneous PNS has been widely studied including with multiple double-blinded randomized controlled trials (RCTs) for a variety of pain conditions [[28], [29], [30], [31], [32], [33]], evaluation of percutaneous PNS of the medial branch nerves for the treatment of CLBP has previously been limited to case reports and small single center studies [[34], [35], [36], [37], [38], [39]].

The primary objective of this prospective multicenter clinical trial was to evaluate the responses of CLBP patients receiving percutaneous PNS of the medial branch nerves. The durability of long-term effects following temporary treatment are characterized in this report. This study reports follow up data through 14 months (*i.e.,* 12 months after the 2-month PNS treatment) on key outcomes including sustained pain relief, back pain-related disability, pain interference, and opioid consumption in the largest cohort to date of CLBP patients with refractory LBP treated with percutaneous PNS.

## 2 Methods

This study received institutional review board (IRB) approval from the Quorum Review IRB (now Advarra), Seattle, WA and the principles of the Declaration of Helinsky were followed. Participants with chronic LBP confined to the lumbar spinal region without radiation to the extremities were screened for eligibility in this prospective, multicenter case series study. Written, informed consent was obtained from each participant prior to completion of any study procedures and the study was registered on June 7, 2017 ​at ClinicalTrials.gov (NCT03179202). This clinical trial was initially conducted under an FDA Investigational Device Exemption (IDE), but following FDA 510(k) clearance of the device during the study, the study was converted to a post-market study following communication with the FDA via an amendment under the same IRB approval. An earlier publication described the results of this trial through the 2-month (end-of-treatment) primary endpoint [40]. This publication describes the long-term results of this clinical trial in all participants (previously, all participants had been followed through 6 months post-treatment) through 12 months after PNS lead removal (14 months total).

### 2.1 Eligibility criteria

Participants were required to have had axial CLBP (*i.e.,* pain confined to the lumbar region with an average pain score ≥4 using a 0–10 scale) for at least 12 weeks duration. Additional inclusion criteria included previous failure of at least two different categories of LBP treatments (*e.g.*, medications, physical therapy, injections, radiofrequency ablation), and stable analgesic medication usage for 4 weeks. Key exclusion criteria included radicular leg pain, prior lumbar surgery, lumbar anesthetic injections within 3 months of enrollment except for diagnostic medial branch blocks, lumbar radiofrequency ablation within 6 months, lumbar scoliosis, ongoing secondary gain, known allergy to adhesives, body mass index (BMI) ​≥ ​40, moderate depression as evidenced by a score >20 on the Beck Depression Inventory (BDI-II), and conditions that are contraindicated with PNS placement such as having a pacemaker. A baseline physical exam was conducted to confirm the participant's eligibility and review LBP-related history including imaging and prior treatments. Apart from the eligibility criteria, there were no specific requirements for pain of a specific etiology, and participants with various sources of axial pain were eligible for inclusion (*e.g.*, degenerative disc disease, facetogenic pain, nonspecific back pain). Following the screening visit, eligible participants completed a 7-day written diary of daily average back pain intensity (Brief Pain Inventory, Question #5, BPI-5). Final eligibility required a mean score ≥4 to qualify for PNS lead implantation.

### 2.2 Percutaneous PNS lead implantation

**Fig. 1:**
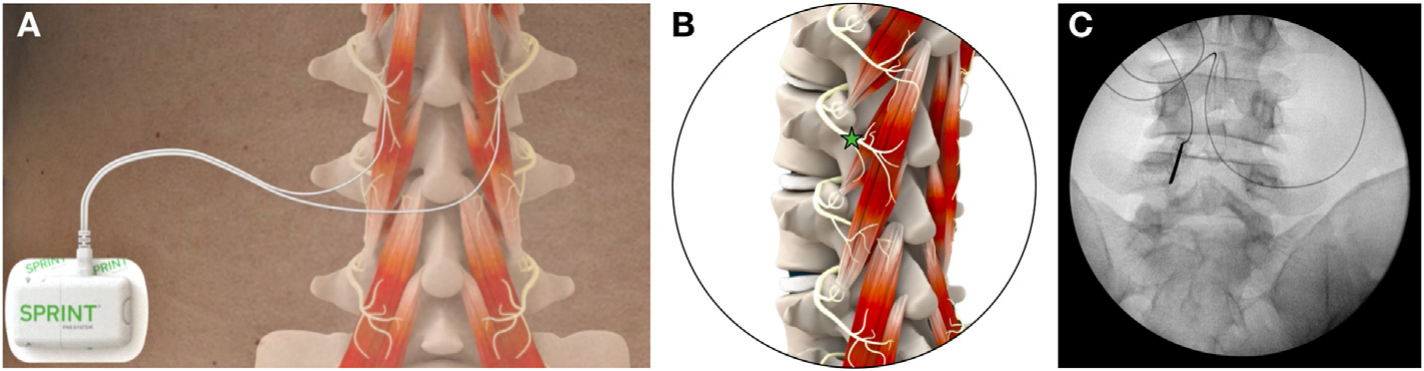
**Percutaneous PNS lead implantation targeting the lumbar medial branches of the dorsal rami. A)** Subjects received percutaneous PNS (SPRINT PNS System), which included a body-worn stimulator connected to two percutaneous fine-wire, coiled leads implanted targeting the lumbar medial branches of the dorsal rami for up to 60 days. **B)** An oblique view of paraspinal anatomy demonstrates the target location for percutaneous PNS lead implantation, the lumbar medial branch nerves over lamina as they course medial and inferior to the facet joint and innervate the multifidus muscles. **C)** An anteroposterior (AP) fluoroscopic image of PNS introducer needle, demonstrating lead implantation targeting the lumbar medial branch nerves over lamina, medial and inferior to the facet joint.

Percutaneous fine-wire, open-coil leads (SPRINT® PNS System MicroLead™, SPR Therapeutics, Inc, Cleveland, OH) were implanted bilaterally to target the medial branch of the dorsal ramus at the vertebral level in the center of the participant's region of pain. All lead implantation procedures were conducted under ultrasound and/or fluoroscopic guidance. The PNS leads were implanted approximately 2 ​cm lateral from midline at an angle approximately 90° to the skin to a depth of approximately 5 ​cm, targeting the medial branch nerves as they course over lamina (*i.e.*, electrode tip was typically positioned approximately 0.5 ​cm off of the lamina), medial and inferior to the facet joint (Fig. 1B, Fig. 1C). Successful implantation and stimulation of the medial branch nerves was confirmed by visualization of activation of the lumbar multifidi under ultrasound and participants' reports of comfortable sensations in the region of axial CLBP. Percutaneous PNS lead deployment was completed by removal of the introducer needle, leaving the percutaneous leads in the tissue, which were typically secured with surgical glue and a waterproof dressing. The percutaneous PNS leads were connected to body-worn pulse generators (SPRINT® PNS System, SPR Therapeutics; Fig. 1A), which were programmed for each participant. Participants were permitted to adjust stimulation intensity levels within a customized range of stimulation settings that generated comfortable, cyclical activation of the multifidi (stimulation parameters: frequency: 12 ​Hz; duty cycle: 50%; amplitude range: 0–30 ​mA; pulse duration range: 10–200 μs). Participants were instructed to use percutaneous PNS for at least 6 ​h per day and up to 12 ​h per day for 60 days. At the end of the 60-day percutaneous PNS treatment, the open-coil leads were withdrawn using gentle traction (*i.e.*, without surgery in a routine clinic visit). During the 2-month treatment period, participants were encouraged to continue most normal activities and returned to the study site for regular follow up visits. Following PNS lead removal, participants completed long-term follow up visits (*i.e.,* up to 12 months after the 2-month PNS treatment, 14 months total) to explore the durability of effects following percutaneous PNS.

### 2.3 Study outcome measures

As reported previously, the study's primary endpoint was pre-defined as the proportion of participants who experienced clinically significant reductions in axial CLBP, as determined by ​≥ ​30% reduction in the Brief Pain Inventory Question #5 (“average pain intensity”) at the end of the 2-month treatment (*i.e.,* average of last week of PNS) compared to baseline (*i.e.,* average of a week prior to the start of PNS). Participants recorded their daily scores and analgesic medication usage in weekly diaries prior to each study visit at 3-month intervals through 12 months after lead removal.

Functional, secondary outcomes were assessed via validated survey instruments at baseline and follow up visits and included: back pain-related disability (Oswestry Disability Index, ODI), pain interference (Brief Pain Inventory, Question #9, BPI-9), patient global impression of change (PGIC), depression (BDI-II), and health-related quality of life (RAND-36). A clinically meaningful improvement in disability was defined as a ≥10-point reduction in ODI and a clinically meaningful improvement in pain interference was defined as a ≥30% BPI-9. Outcomes were also analyzed for follow up visits after end of treatment with PNS to explore the durability of effects following percutaneous PNS. Adverse events were collected throughout the duration of the clinical study.

### 2.4 Statistical analysis and data handling

Statistical data analyses are reported as observed, without imputation for missing data. Data are analyzed with one-way ANOVA with a p ​= ​0.05 level of significance and included post hoc Tukey-Kramer adjustment for multiple comparisons. Data reported are shown as mean (standard deviation), unless otherwise stated.

## 3 Results

### 3.1 Baseline values & demographics

Participants reported an average 16.0 (SD 13.0) year duration of CLBP and were on average 56.3 (SD 13.5) years of age (n ​= ​74). Other key participant demographics are shown in Table 1. A detailed subject participation flow diagram has been previously presented [40]. Of the 81 eligible patients who started PNS treatment, 7 participants were lost to follow up and 74 participants completed PNS treatment, providing data for evaluation at the Primary Endpoint (2 months after start of PNS). Twelve additional participants were lost to follow up throughout participation in the long-term follow up visits, with 62 participants completing the final follow up visit at 14 months.

**Table 1:** Baseline info and demographics.

Participant Demographics (n ​= ​74)	
Age (years)	56.3 (13.5)
Body Mass Index (BMI)	29.4 (4.6)
LBP Duration (years)	16.0 (13.0)
Sex (% Female)	53%
Work Status at Baseline	
Currently Working	45%
Retired (Not due to Health)	28%
Disabled due to Back Pain	8%
Unemployed	5%
Other (*e.g.,* Homemaker, On Leave of Absence, Student, Other)	12%
Previously Failed LBP Treatments:	
Non-opioid Analgesics	97%
Physical Therapy	89%
Opioid Analgesics	67%
TENS	65%
Anesthetic or Steroid Injections	57%
Epidural Injections	46%
Radiofrequency Ablation	23%
*Results shown as Mean (SD) unless otherwise stated.*	

At baseline, participants rated their CLBP as moderate to severe with scores of 6.1 (1.2) for average pain intensity (BPI-5) and 7.6 (1.2) for worst pain intensity (BPI-3). Participants reported back pain-related disability that was moderate to severe, with an average baseline score of 38.3 (12.5) on ODI and substantial interference of pain on daily activities with a score of 5.6 (2.1) on BPI-9. Table 1 shows the prior LBP treatments participants had received that failed to provide sufficient relief prior to enrollment in this study, which included: non-opioid analgesics (97% of participants), physical therapy (89%), opioid analgesics (67%), transcutaneous electrical nerves stimulation (TENS, 65%), chiropractic manipulation (61%), lumbar anesthetic or corticosteroid injections (57%), epidural injections (46%), and radiofrequency ablation (23%). The average number of failed therapies was 5.3 across all subjects. Participants were most commonly diagnosed as having lumbar spondylosis (37%) or degenerative disc disease (32%); however 28% had no known etiology of pain or were listed as having non-specific CLBP. Percutaneous PNS leads were implanted at the spinal level in the middle of the participant's most painful low back region. Leads were most commonly implanted at the L4 (44%) or L5 (42%) spinal levels and 91% (n ​= ​67/74) of participants received two leads placed bilaterally flanking the spinous process at that level (*i.e.,* one lead on each side).

### 3.2 Results after 2 ​Months of percutaneous PNS

As reported previously [40], 73% of participants (n ​= ​54/74) were defined as responders and reported clinically meaningful (≥30%) reductions in average back pain intensity (BPI-5) after the 2-month percutaneous PNS treatment (Primary Endpoint), which corresponded to an average 58% reduction in BPI-5 among responders. The finding that a majority of participants successfully responded to PNS is further supported by consistent findings in a hypothetical sensitivity analysis (using imputation to failure for missing data), where 67% (n ​= ​54/81) of the participants would be defined as responders at the Primary Endpoint. After 2 months of percutaneous PNS, clinically meaningful reductions in CLBP-related disability (as measured by ODI) were reported by 73% (n ​= ​53/73) of participants, with an average 21-point ODI reduction among responders. Clinically meaningful improvements in pain interference were reported by 73% (n ​= ​53/73) of participants and among responders the average reduction in pain interference was 67% at the end of the 2-month percutaneous PNS treatment. Table 2 shows the mean scores for all participants for pain intensity, disability, and pain interference. All other prospectively defined endpoints, including patient global impression of change (PGIC), depression (BDI-II), and health-related quality of life (RAND-36), showed clinically meaningful and/or substantial improvement with the 2 month percutaneous PNS treatment and were reported in the previous publication [40].

**Table 2:** Statistically significant reductions in pain intensity, disability, and pain interference through 12 months after percutaneous PNS lead removal (14 months total).

Average Pain Intensity (BPI-5)			
Timepoint	Mean	SEM	*p*
Baseline (n ​= ​74)	6.08	0.14	*-*
2 ​Months (n ​= ​74)	3.59	0.22	*<.0001*
5 ​Months (n ​= ​70)	3.59	0.25	*<.0001*
8 ​Months (n ​= ​66)	3.89	0.26	*<.0001*
11 ​Months (n ​= ​62)	4.04	0.30	*<.0001*
14 ​Months (n ​= ​62)	3.87	0.27	*<.0001*
Oswestry Disability Index (ODI)			
Timepoint	Mean	SEM	*p*
Baseline (n ​= ​74)	38.33	1.45	***-***
2 ​Months (n ​= ​74)	23.30	1.50	*<.0001*
5 ​Months (n ​= ​70)	25.01	1.76	*<.0001*
8 ​Months (n ​= ​65)	27.46	1.86	*<.0002*
11 ​Months (n ​= ​61)	28.74	2.03	*0.002*
14 ​Months (n ​= ​62)	28.86	1.97	*0.002*
Pain Interference (BPI-9)			
Timepoint	Mean	SEM	*p*
Baseline (n ​= ​74)	5.61	0.88	*-*
2 ​Months (n ​= ​73)	2.72	0.24	*<.0001*
5 ​Months (n ​= ​70)	3.04	0.28	*<.0001*
8 ​Months (n ​= ​65)	3.32	0.30	*<.0001*
11 ​Months (n ​= ​60)	3.68	0.32	*<.0001*
14 ​Months (n ​= ​61)	3.63	0.29	*<.0001*

### 3.3 Durability of patient-reported outcomes following temporary, percutaneous PNS

**Fig. 2:**
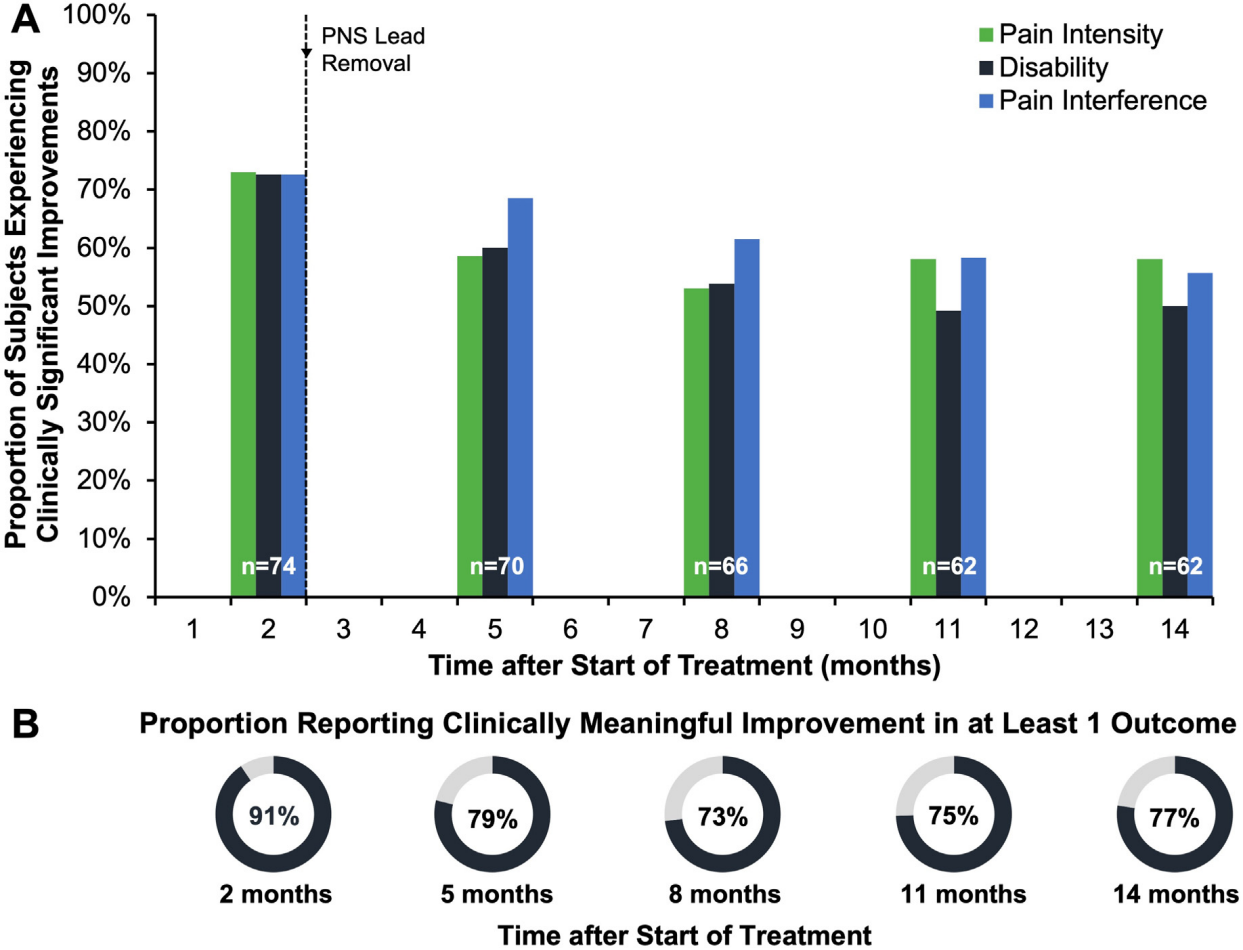
**Proportion of subjects experiencing clinically significant reductions in Pain Intensity, Disability, and Pain Interference through 14 months after percutaneous PNS. A)** A majority of participants experienced clinically significant reductions in prospectively-defined endpoints of average pain intensity (≥30% reduction BPI-5), disability (≥10-point reduction Oswestry Disability Index, ODI), and/or pain interference (≥30% reduction BPI-9). Improvements were sustained long-term following lead removal. Colored bars represent proportions of subjects reporting clinically significant improvements for each outcome over time after start of PNS treatment. **B)** A majority of subjects reported clinically meaningful improvement in at least one outcome over time, with 77% (n ​= ​48/62) reporting clinically significant improvement at 14 months.

**Fig. 3:**
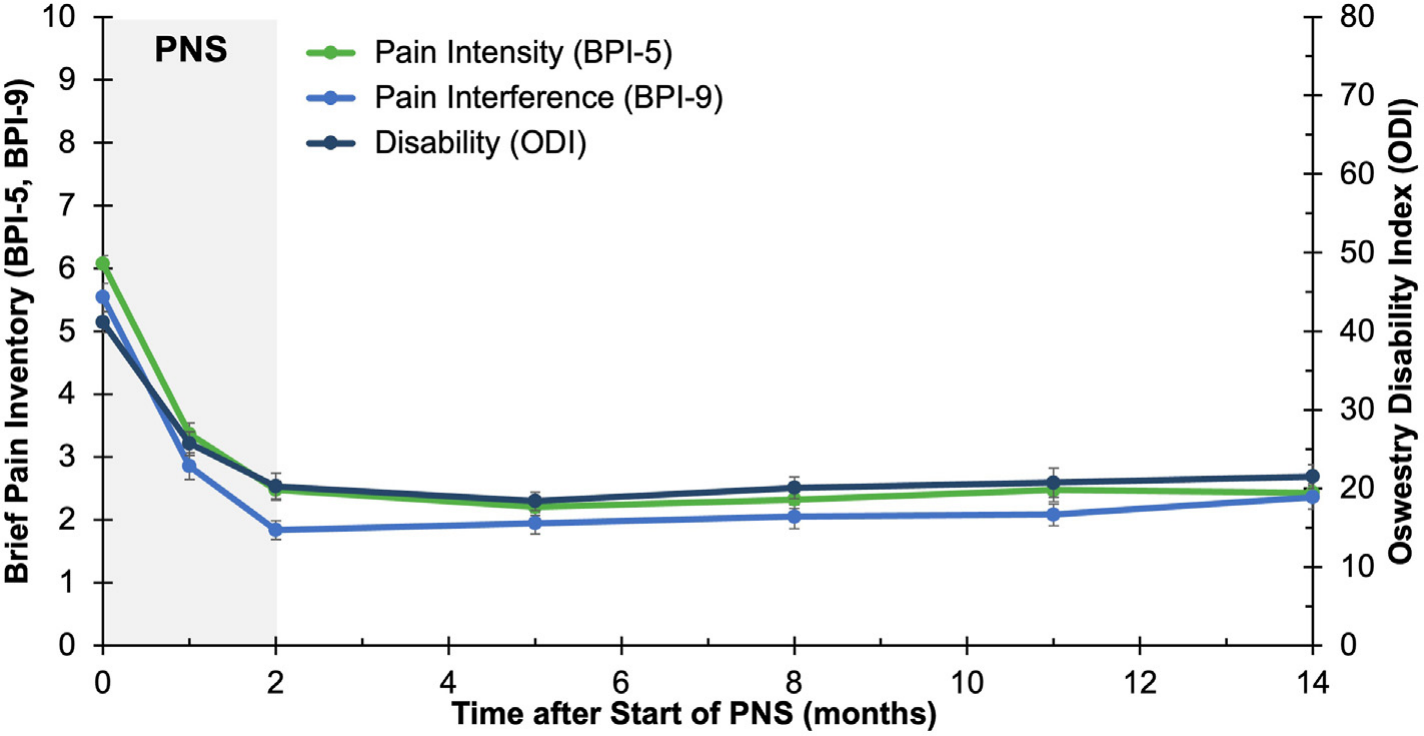
**Sustained improvements among responders following percutaneous PNS.** Clinically meaningful reductions in average pain intensity BPI-5), disability (Oswestry Disability Index, ODI), and pain interference (BPI-9) were sustained long-term among responders (from Fig. 2) through the 14-month duration of follow up. Data shown as mean ​± ​SEM.

Statistically significant reductions in each of the key outcome measures, average pain intensity (BPI-5), disability (ODI), and pain interference (BPI-9), were sustained through 12 months after percutaneous PNS lead removal (14 months total; Table 2, ANOVA, p ​< ​0.05, *post hoc* Tukey-Kramer adjustment for multiple comparisons). A majority of participants reported clinically meaningful reductions in pain intensity, disability, and/or pain interference through 14 months after start of percutaneous PNS (Fig. 2A). The proportions of participants experiencing clinically meaningful improvement in each outcome (BPI-5, ODI, or BPI-9) was 73%, 73%, and 73%, respectively, after 2 months of PNS, and was 59%, 60%, and 69% at 5 months; 53%, 54%, and 62% at 8 months; 58%, 49%, and 58% at 11 months; and 58%, 50%, and 56% at 14 months. Fig. 3 shows the sustained improvements seen among responders at each timepoint through 14 months after start of percutaneous PNS for average pain intensity, disability, and pain interference.

The proportion of participants experiencing clinically meaningful improvement in at least 1 of the 3 outcomes (BPI-5, ODI, or BPI-9; 91% after 2 months of PNS) was 79% at 5 months, 73% at 8 months, 75% at 11 months, and 77% at 14 months (Fig. 2B). The durability of relief with PNS was also confirmed in a hypothetical sensitivity analysis (using imputation to failure for missing data), where a majority, 59% (n ​= ​48/81) were found to have had clinically meaningful improvement in at least one outcome. The proportion of participants experiencing clinically meaningful improvement in two or more outcomes in long-term follow up (77% at 2 months) was 63% at 5 months, 60% at 8 months, 59% at 11 months, and 58% at 14 months.

**Fig. 4:**
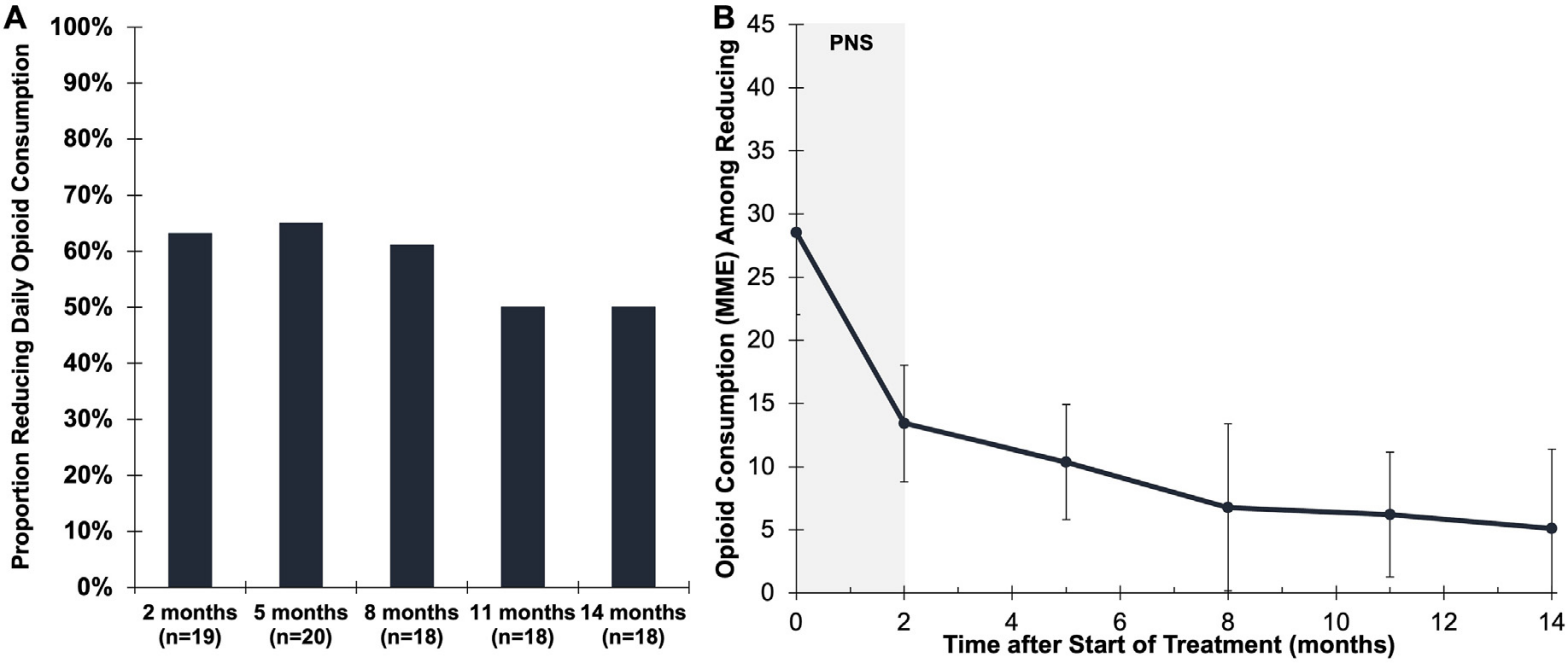
**Reductions in opioid analgesic consumption with percutaneous PNS among those taking opioids at baseline. A)** A majority of subjects taking opioids at baseline (n ​= ​20) reported reductions in opioid consumption in the months following percutaneous PNS. **B)** Among those reducing opioid analgesic consumption, the average consumption reduced from 28.5 ​mg morphine equivalent (MME) at baseline to 13.4 MME after 2 months of PNS, and was further reduced to 5.1 MME at 14 months. Data shown as mean ​± ​SEM.

Most participants who reported taking opioids at baseline (75%, n ​= ​15/20) also reported reductions in opioid consumption in the months after PNS. The reductions in opioid consumption were sustained over the 12-month follow up period after PNS lead removal. Among those reducing opioid analgesic consumption, the average consumption reduced from 28.5 ​mg morphine equivalent (MME) at baseline to 13.4 MME after 2 months of PNS, and was further reduced to 5.1 MME at 14 months (Fig. 4). The proportion of participants who had been taking opioids at baseline and later reported cessation, or no opioid consumption, at each timepoint was 21% at 2 months, 20% at 5 months, 17% at 8 months, 28% at 11 months, and 17% at 14 months after start of PNS.

Twelve months after PNS lead removal, a majority of participants reported that they were “Satisfied” or “Very Satisfied” with the pain relief that they received following treatment (57%, n ​= ​35/61) in a Subject Satisfaction Survey. A majority of participants also “Agreed” or “Strongly Agreed” that they would recommend this treatment to a friend with low back pain (69%, n ​= ​42/61) and preferred using stimulation to taking pain medications (72%, n ​= ​44/61). If it had been available, 75% (n ​= ​46/61) “Agreed” or “Strongly Agreed” that they would have pursued this treatment earlier in their care pathway.

### 3.4 Adverse events

No serious, unanticipated study-related adverse events occurred. Adverse events related to use of temporary, 60-day percutaneous PNS in this study were previously reported in the first publication of this clinical trial [40], in which data for the use of PNS was complete through 6 months after lead removal. All study-related adverse events were non-serious (mild or moderate) and followed to resolution. The most common adverse events were mild skin irritation or pruritis at the bandage or stimulator's mounting pad. No additional device-related adverse events were reported for these subjects through the follow up period.

## 4 Discussion

This report describes the long-term results (*i.e.,* more than 1 year follow up) from the largest cohort of CLBP participants studied to date in a prospective multicenter clinical trial of 60-day percutaneous medial branch PNS. These findings demonstrate durable improvements in pain intensity, back pain-related disability, and pain interference with function and daily activities following minimally invasive, temporary percutaneous PNS of the medial branch nerves. The clinically and statistically significant reductions in pain, disability, and/or pain interference demonstrate the important clinical benefits provided by this short-term, 60-day treatment for patients with substantial, disabling CLBP (average 16-year duration, average 6.1 pain score at baseline, and average 38-pt ODI score at baseline) who were recalcitrant to prior therapies. Further, this trial's findings corroborate the results of prior smaller, single-center case series on a larger scale. While an earlier publication reported on this trial's primary endpoint results, this report is the first to fully document the complete long-term follow up results, demonstrating the durability of positive patient-reported outcomes following temporary, 60-day percutaneous PNS of the medial branches for CLBP.

In addition to the long-term clinically meaningful and statistically significant reductions in average back pain intensity (BPI-5), pain relief was also accompanied by reports of meaningful, commensurate improvements in back pain-related disability (ODI) and/or pain interference (BPI-9), which were sustained throughout the 12-month follow up period. Percutaneous PNS also produced substantial improvements in PGIC, depression, health-related quality of life and other patient-centric outcomes [40]. The clinically meaningful improvements in patient-centric outcomes substantiate results from prior studies of percutaneous PNS for CLBP [34,35,41,42] by showing long-term improvements in patients refractory to multiple other treatments. Given the temporary, 60-day approach and the potential clinical benefit, percutaneous PNS offers a treatment option appropriate for introduction earlier in the treatment continuum, especially when it can obviate the need for more destructive interventions whose effects may wane over time, such as radiofrequency ablation, surgery, or permanently implanted neurostimulation systems.

No serious or unanticipated study-related adverse events were reported. The most common study-related adverse events reported by participants in this trial were mild skin irritation and pruritis. Percutaneous PNS, including the unique open-coil lead that was specifically developed for temporary, percutaneous placement in the periphery, was designed to enable a safe, effective, non-opioid, neuromodulation treatment option earlier in the treatment continuum.

### 4.1 Mechanism of action (MOA)

As described in the *Introduction*, a large body of evidence exists that a substantial proportion of CLBP involves sensitization of neural processes in the central nervous system (*i.e.,* brain and spinal cord), which result in downstream signaling changes that negatively impact the periphery (*i.e.*, arthrogenic inhibition), amplifying pain in a feed-forward loop of worsening pain and disability. In one study, Förster et al. found that 31% of patients with axial LBP reported likely or possible “neuropathic” or “nociplastic” symptoms on painDETECT despite no identifiable nerve injury, suggesting central sensitization [43]. Patients with CLBP often describe symptoms of central sensitization, such as hypersensitivity to normal stimuli or pain long after an injury has healed [11,[44], [45], [46], [47]]. The literature also shows that effective pain treatments are correlated with a reversal of central pain processing abnormalities [11,48].

A centrally-mediated, neuromodulatory MOA is proposed to underlie the sustained analgesic effects and improvements in functional outcomes (*e.g.,* pain-related disability and pain interference with activities of daily living) that followed pain relief produced by temporary, percutaneous PNS. This mechanism, peripherally-induced reconditioning of the central nervous system, was first proposed by Deer and colleagues [26]. It is believed that percutaneous PNS, when applied to medial branch nerves for the treatment of CLBP, generates robust activation of peripheral nerve efferent fibers that induce multifidus activation, and afferent fibers that directly activate large diameter proprioceptive fibers in the medial branch, as well as through indirect activation of healthy proprioceptive signals following contraction of the multifidus. When combined, the design of the percutaneous PNS system, stimulation parameters, and lead configuration produce robust and focal activation of the medial branch nerve [26]. Further, the focal specificity of the neural signals produced with percutaneous PNS, which specifically targets the area of pain, is important to produce the activity-dependent cortical remapping needed to reverse the central features of chronic pain and provide sustained relief [26].

The durable, long-term effects of short-term percutaneous PNS treatment (*i.e.,* clinically and statistically significant improvements in outcomes 12 months after a 2-month PNS treatment) reported by a majority of participants in this prospective clinical trial help to further corroborate the centrally-mediated MOA. Given the prolonged effects and durability of relief provided by this short-term PNS treatment, reconditioning of central sensitization processes is believed to be responsible for the maintenance of relief following the removal of percutaneous PNS leads. Since a temporary peripheral insult or injury often precedes the initiation of centrally-mediated processes, it is plausible that a temporary stimulation approach to restore peripheral inputs (*i.e.,* percutaneous PNS) could provide an opportunity to recondition the centrally-mediated pain state, setting patients on a path to improvement and recovery, without requiring more invasive neuromodulatory or destructive analgesic interventions that themselves are associated with habituation [27]. This MOA is further supported by the finding that participants who had failed prior analgesic therapies, such as injections or radiofrequency ablation (which aim to block or remove sensory signals), experienced pain relief with PNS (which aims to address the underlying, centrally-mediated changes in pain processing by the generation of focal and robust neural signals coming from the region of pain in the periphery).

### 4.2 Study limitations

This study did not randomize participants to placebo or another intervention, as percutaneous PNS has been previously demonstrated to provide statistically significant pain relief versus control in 5 published randomized controlled trials [[28], [29], [30], [31], [32], [33]]. This clinical trial was designed as a prospective, multicenter case series study to explore the effects of percutaneous PNS among patients with CLBP refractory to conventional non-surgical treatments. The results of this prospective, multicenter clinical trial are consistent with the results of previous clinical trials (including multiple Level 1, placebo-controlled RCTs) [[28], [29], [30], [31], [32], [33]]. While participants with a variety of CLBP diagnoses (*e.g.,* discogenic back pain, lumbar spondylosis), as well as many with no formal diagnosis of the source of CLBP (e.g., non-specific pain), were enrolled in this trial, there were no significant differences between outcomes with PNS for these groups. Future studies and publications are expected to further explore the relationship of source of back pain and outcomes with PNS.

### 4.3 Summary

This large, prospective multicenter clinical trial demonstrates the clinical utility of percutaneous PNS when applied to the medial branch nerves for the treatment of chronic axial low back pain recalcitrant to an average of 5.3 non-surgical therapies. Temporary, 60-day percutaneous PNS treatment produced clinically meaningful improvements in pain, disability, and/or pain interference long-term for a majority of participants through the entire 14 month follow up period without requiring permanent system implantation. The safety profile of percutaneous PNS suggests that this innovative, minimally invasive approach is safe and effective for CLBP and that well-selected patients are likely to experience significant benefit. Given the minimally invasive nature of percutaneous PNS and the significant benefits experienced by participants, percutaneous PNS should be considered as a first-line neurostimulation treatment for use early in the care pathway for patients with axial CLBP.

## Funding

This study was sponsored by SPR Therapeutics, Inc.

## Declaration of competing interest

The authors declare the following financial interests/personal relationships which may be considered as potential competing interests:

Christopher Gilmore, Mehul Desai, Sean Li, Michael DePalma, Timothy Deer, and Steven Cohen reports financial support was provided by SPR Therapeutics Inc. Christopher Gilmore, Mehul Desai, Sean Li, Michael DePalma, Timothy Deer, and Steven Cohen reports a relationship with SPR Therapeutics Inc that includes: consulting or advisory, speaking and lecture fees, and travel reimbursement. Joseph Boggs and Meredith McGee has patent issued to SPR Therapeutics, Inc.

Drs Gilmore, Desai, Hopkins, Li, DePalma, and Deer are physician investigators with clinical research sponsored by SPR Therapeutics. Drs Gilmore, Desai, Li, Deer, and Cohen are consultants for SPR, and Drs Desai and Deer have equity ownership in SPR Therapeutics. Drs McGee and Boggs are employees of SPR Therapeutics with equity ownership.
